# Cronic creatine supplementation and physical exercisereduces on oxidative stress in Wistar rats

**DOI:** 10.1186/1753-6561-8-S4-P9

**Published:** 2014-10-01

**Authors:** Suênia Porpino, Naiane Ferraz, Matheus Monteiro, Thyago Queiroz, Renata Travassos, Valdir Braga

**Affiliations:** 1 Universidade Federal da Paraíba, Paraíba, Brazil

## Background

It has been reported that creatine could act as an antioxidant preventing increase oxidative stress. We investigated whether creatine supplementation and physical exercise could affect oxidative stress in Wistar rats.

## Methods

Twenty-four rats were divided into three groups: control (CO, n = 8), creatine supplemented (CR, n = 8; 0.5g creatine/kg/day, by gavage for 4 weeks) and exercise (EX, n = 8; swimming for 1 h/d, for 4 weeks). Oxidative stress was measured by tiobarbituric acid reactive species assay (TBARS) in serum, heart, kidney, liver, gastrocnemius muscle and nervous system (cortex, midbrain, cerebellum and brainstem).

## Results and conclusions

CR reduced oxidative stress in serum (10.7 ± 1.9 vs. 6.9 ± 1.8; nmol/g, p < 0.05), heart (6.9 ± 1.4 vs. 2.9 ± 1.0 nmol/g, p < 0.05), kidney (7.6 ± 1.5 vs.4.7 ± 0.6 nmol/g, p < 0.05) and liver (4.2 ± 0.4 vs. 3.5 ± 0.3 nmol/g, p < 0.05) as well as in the midbrain (12 ± 1.5 vs. 6.7 ± 1.0 nmol/g, p < 0.05) and cerebellum (11.1 ± 2.5 vs 10.2 ± 2.6 nmol/g, p < 0.05) compared to control. EX reduced oxidative stress in serum (10.7 ± 1.9 vs. 7.7 ± 2.0 nmol/g, p < 0.05), kidney (7.6 ± 1.5 vs. 4.3 ± 0.7 nmol/g, p < 0.05) and liver (4.2 ± 0.4 vs. 3.1 ± 0.5 nmol/g, p < 0.05) but not in heart and gastrocnemius muscle. In addition, EX reduced oxidative stress in the cortex (13.1 ± 2.7 vs. 7.1 ± 1.1 nmol/g, p < 0.05) and midbrain (12 ± 1.5 vs. 4.1 ± 1.8 nmol/g, p < 0.05). In conclusion, creatine and physical exercise reduced oxidative stress in Wistar rats.
